# *Fusarium sporotrichioides* Produces Two HT-2-α-Glucosides on Rice

**DOI:** 10.3390/toxins16020099

**Published:** 2024-02-10

**Authors:** Thomas Svoboda, Roman Labuda, Michael Sulyok, Rudolf Krska, Markus Bacher, Franz Berthiller, Gerhard Adam

**Affiliations:** 1 Institute of Microbial Genetics, Department of Applied Genetics and Cell Biology, University of Natural Resources and Life Sciences Vienna, Konrad-Lorenz-Str. 24, 3430 Tulln, Austria; markus.bacher@boku.ac.at; 2 Unit of Food Microbiology, Institute of Food Safety, Food Technology and Veterinary Public Health, Department for Farm Animals and Veterinary Public Health, University of Veterinary Medicine Vienna, Veterinaerplatz 1, 1210 Vienna, Austria; roman.labuda@vetmeduni.ac.at; 3 Research Platform Bioactive Microbial Metabolites (BiMM), Konrad Lorenz Strasse 24, 3430 Tulln, Austria; 4 Institute of Bioanalytics and Agro-Metabolomics, Department of Agrobiotechnology (IFA-Tulln), University of Natural Resources and Life Sciences Vienna, Konrad Lorenz Strasse 20, 3430 Tulln, Austria; michael.sulyok@boku.ac.at (M.S.); rudolf.krska@boku.ac.at (R.K.); franz.berthiller@boku.ac.at (F.B.); 5 Austrian Competence Centre for Feed and Food Quality, Safety and Innovation FFoQSI GmbH, Konrad-Lorenz-Strasse 20, 3430 Tulln, Austria; 6 Institute of Chemistry of Renewable Resources, Department of Chemistry, University of Natural Resources and Life Sciences Vienna, Konrad-Lorenz-Strasse 24, 3430 Tulln, Austria

**Keywords:** mycotoxins, glycosylation, Fusarium, HT-2-alpha-glucoside

## Abstract

*Fusarium* is a genus that mostly consists of plant pathogenic fungi which are able to produce a broad range of toxic secondary metabolites. In this study, we focus on a type A trichothecene-producing isolate (15-39) of *Fusarium sporotrichioides* from Lower Austria. We assessed the secondary metabolite profile and optimized the toxin production conditions on autoclaved rice and found that in addition to large amounts of T-2 and HT-2 toxins, this strain was able to produce HT-2-glucoside. The optimal conditions for the production of T-2 toxin, HT-2 toxin, and HT-2-glucoside on autoclaved rice were incubation at 12 °C under constant light for four weeks, darkness at 30 °C for two weeks, and constant light for three weeks at 20 °C, respectively. The HT-2-glucoside was purified, and the structure elucidation by NMR revealed a mixture of two alpha-glucosides, presumably HT-2-3-*O*-alpha-glucoside and HT-2-4-*O*-alpha-glucoside. The efforts to separate the two compounds by HPLC were unsuccessful. No hydrolysis was observed with two the alpha-glucosidases or with human salivary amylase and *Saccharomyces cerevisiae* maltase. We propose that the two HT-2-alpha-glucosides are not formed by a glucosyltransferase as they are in plants, but by a trans-glycosylating alpha-glucosidase expressed by the fungus on the starch-containing rice medium.

## 1. Introduction

**Figure 1:**
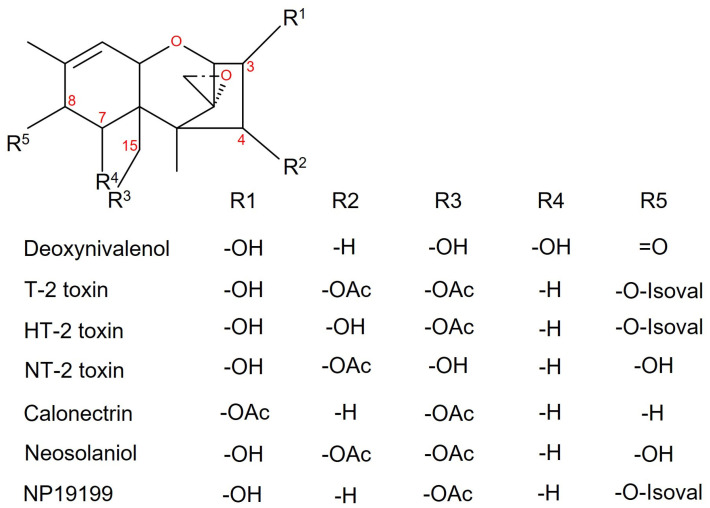
Structures of selected trichothecenes.

Trichothecenes are toxic secondary metabolites which are produced predominantly by species of *Fusarium*, *Stachybotrys*, or *Trichoderma* [1]. The epoxy-trichothecene core structure of these sesquiterpenoids contains a 12, 13 epoxide group which is essential for toxicity [2]. Trichothecenes can be divided into type A and type B trichothecenes depending on the substituent on C8. While type B trichothecenes (e.g., deoxynivalenol, DON, see Figure 1) have a keto group at C8, type A trichothecenes are not oxygenated at this position, or they possess an oxygen function other than keto, typically a hydroxy group that can be esterified.

In the biosynthesis of type B trichothecenes, the product of the *Fusarium graminearum* (*Fg*) *TRI1* gene introduces hydroxy groups at both C7 and C8 of calonectrin (R^4^ and R^5^ in Figure 1) [3]. In *Fusarium sporotrichioides* (*Fs*), Tri1 hydroxylates only C8 of 3, 4, 15-triacetoxyscirpenol [4]. In the next step, an isovaleryl group is attached by the gene product of *FsTRI16*, yielding 3-acetyl T-2-toxin. This compound is translocated across the plasma membrane and deacetylated (C3-OH, R^1^) by the *TRI8* gene product, resulting in T-2 toxin [1]. Further deacetylation (C4-OH, R^2^) leads to hydrolyzed T-2 toxin (HT-2 toxin). In addition to these main products, many strains produce other closely related structures known as NT-2-toxin (https://pubchem.ncbi.nlm.nih.gov/compound/Toxin-NT-2, accessed on 1 January 2024) and 4-deoxy-T-2-toxin (NP19199, https://pubchem.ncbi.nlm.nih.gov/compound/102514951, accessed on 1 January 2024). NP19199 is similar to T-2 toxin but lacks the hydroxylation at C4 (R^2^), due to the omission of the *TRI13* dependent step [5] (see Supplementary Figure S1). NT-2 toxin has a hydroxy group at C15 (R^5^) while all the other residues are shared with neosolaniol [6]. In Figure 1, the structures of the selected trichothecenes are shown.

T-2 toxin is highly toxic for rats, mice, pigeons, and guinea pigs, with an LD_50_ in the range of 1–14 mg/kg body weight upon intravenous, intragastric, subcutaneous, intraperitoneal, or intratracheal application [7]. For humans, a tolerable daily intake (TDI) of the sum of the T-2 and HT-2 toxins has been set at 0.1 µg/kg body weight by the European Food Safety Authority (EFSA) [8]. Currently, no legally binding maximum level for the T-2 and HT-2 toxins has been enacted for food commodities in Europe [9], but the addition of a sum parameter for the T-2 and HT-2 toxins is expected by July 2024. With regard to DON, in the toxicological risk assessment, a group parameter for DON, acetylated DON, and DON-glucoside has been established by the EFSA, as the derivatives can be hydrolyzed back to the parental toxin in the intestines [10].

Several microbial biotransformation products of trichothecenes, like hydroxylation of the isovaleryl-side-chain of T-2 toxin (C3′-OH or C4′-OH) [11] or de-epoxidation [12,13] have been described. In wheat, most of the multiple T-2 and HT-2 toxin biotransformation products are formed by glycosylation and further metabolism of the respective glucosides [14].

Plants possess a large number of genes (about 150 in diploid plants) that encode family 1 UDP-glycosyltransferases (UGTs) [15] and for conjugation of small molecules. Some of them are capable of detoxifying trichothecenes [16]. The plant UGT family 1 members are inverting enzymes which exclusively produce beta-glucosides of the aglycons with the co-substrate UDP-alpha-D-glucose preferred by most enzymes. The first structurally characterized enzyme (OsUGT79 gene product) acting on DON was expressed in *E. coli* and used to enzymatically generate an HT-2-3-*O*-beta-glucoside reference material [16]. As this enzyme is unable to glycosylate C4-acetylated toxins (e.g., nivalenol, T-2 toxin)—unless three amino acid changes are introduced [17]—another enzyme (HvUGT13248 from barley) was used to generate a T-2-3-*O*-beta-glucoside standard [16].

While T-2 toxin can only be glucosylated at the C3-OH, in HT-2 both the C3 and the C4 hydroxyl groups are available for glycosylation. A double mutant of *F. graminearum* (*tri5*Δ *tri101*Δ), which is unable to produce trichothecenes and to detoxify them by C3-acetylation, attaches glucose to C4-OH when challenged with HT-2 toxin, interestingly forming an alpha-glycosidic linkage [18]. Glycosylation at C4-OH results in decreased toxicity. Busman et al. were the first to report mass spectrometric evidence that a 3-*O*-glucoside of T-2 toxin, as well as of HT-2 toxin, can be produced by *Fusarium sporotrichioides* [19], but they did not determine the anomeric state of glucose in these compounds. In the literature, there is some controversy about whether alpha- or beta-glucosides are present in naturally contaminated grain samples (see discussion). This could be toxicologically relevant if the two types of glucosides are metabolized differently, by alpha- or beta-glucosidases with different localization in the intestinal tract.

In this study, we describe the secondary metabolite pattern of an Austrian *Fusarium sporotrichioides* isolate and assess the impact of different conditions (temperature, light, and upscaling) on the production of T-2, HT-2-toxin, and HT-2-glucoside. Furthermore, the anomeric configuration of the fungus-produced HT-2-glucoside was determined, and the response to the two alpha-glucosidases was tested.

## 2. Results

### 2.1. Secondary Metabolite Profile of Fusarium sporotrichioides 15-39

**Figure 2:**
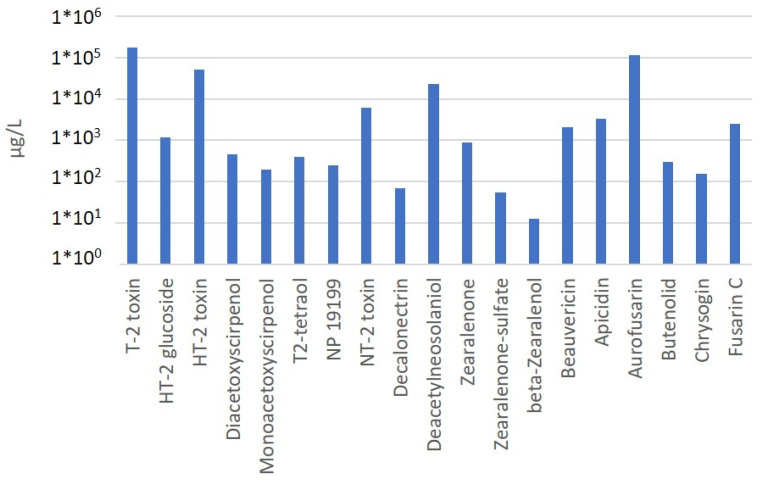
Toxins produced by *Fusarium sporotrichioides* 15-39, indicated as µg/L in the obtained extract (rubofusarin was not quantified due to the lack of a standard for quantification).

The strain used in this study is a *Fusarium sporotrichioides* strain isolated in 2015 from maize in the district of Tulln in Lower Austria. After molecular identification by the sequencing of the translation elongation factor PCR product, this strain was grown on autoclaved rice and the metabolites were extracted. To determine the secondary metabolite profile, a multimethod that was available at that time was used [20]. As shown in Figure 2, different type A trichothecenes were detected.

According to this pilot experiment, in the small-scale culture with 2 g rice, *F. sporotrichioides* 15-39 yielded 177 mg/L T-2 toxin in the extract from the rice, in addition to 51 mg/L HT-2. Interestingly, 1.2 mg/L HT-2-glucoside was also detected, which corresponds to 6 mg HT-2-glucoside per kg rice. Considering the trichothecenes produced, about 68% was T-2 toxin, 20% was HT-2 toxin, and about 0.46% was HT-2-glucoside. This result was based on a retention time and a mass that were identical to those of the available HT-2-3-*O*-beta-glucoside standard and a similar fragmentation pattern. Other compounds which made up more than 1% of the trichothecenes were NT-2 toxin (=15-deacetyl-neosolaniol), with about 2.4%, and 4-deacetyl-neosolaniol (indicated as deacetylneosolaniol in Figure 2), with about 9%, while the remaining detected trichothecenes were below 1%.

**Figure 3:**
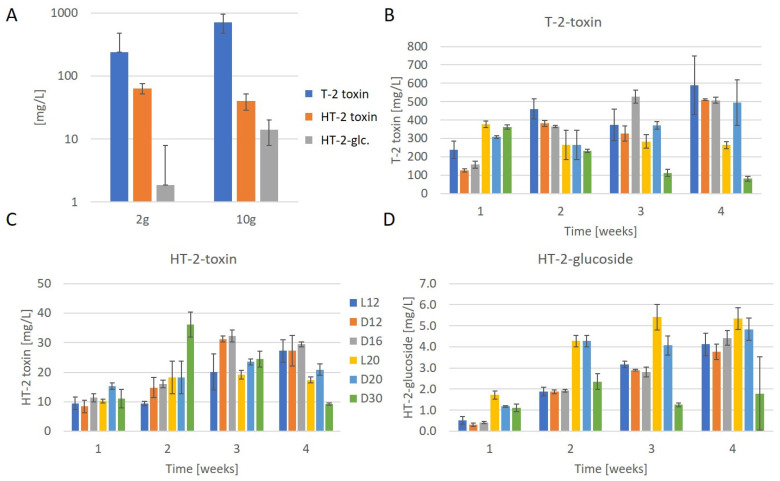
Effect of temperature, light, and incubation duration on toxin production (mg/L extract); (**A**) T-2 toxin, HT-2 toxin, and HT-2-glucoside production on 2 g and 10 g rice after 2 weeks; (**B**) T-2 toxin production over time; (**C**) HT-2-toxin production over time; (**D**) HT-2-glucoside production over time; incubation conditions: L12 = constant light, 12 °C, D12 = darkness, 12 °C; D16 = darkness, 16 °C; L20 = constant light, 20 °C; D20 = darkness, 20 °C; D30 = darkness, 30 °C.

In this first test, an agar plug was used for inoculation. As described in Materials and Methods, the experiment was repeated with 2 g as well as 10 g rice with three replicates, which were inoculated with conidiospores for better reproducibility. The amount of T-2 toxin produced on 2 g rice was on average a 238 mg/L extract, which was slightly higher than that of the first test. Upscaling had no negative effect: on 10 g rice, significantly higher amounts (*p* = 4.1 × 10^−5^) of T-2 toxin (714 mg/L) were observed. HT-2-glucoside was also significantly higher (*p* = 9.3 × 10^−6^) on 10 g rice, with 14 mg/L, compared to 2 g rice, where 2 mg/L was measured. In contrast, on 2 g rice an average HT-2-toxin level of 64 mg/L was detected, whereas on 10 g rice only 40 mg/L was detected (Figure 3A).

### 2.2. Optimization of T-2 Toxin, HT-2 Toxin, and HT-2-Glucoside Production Conditions

To determine the optimal T-2 toxin production conditions, we tested incubation at different temperatures between 12 and 30 °C as well as under constant white light and in darkness. The test was conducted with 10 g rice with three replicates per condition and time point. In Figure 3, the results of the production of T-2 toxin (3B), HT-2 toxin (3C), and HT-2-glucoside (3D) over time are shown.

Our results show that the highest amount of T-2 toxin (on average 589 mg/L) was produced after the incubation of the samples at 12 °C for four weeks under constant light, while 363 mg/L was produced at 30 °C within one week. The amount of T-2 toxin in the samples increased constantly over three weeks only when kept at 16 °C in the dark, while incubation under constant light, as well as that in the dark at 12 °C, showed a decrease between two and three weeks of incubation. While the T-2 toxin production at 20 °C under constant light and at 30 °C in the dark was the highest after one week, with 377 mg/L and 363 mg/L, respectively, the amount of toxin decreased at later time points.

Under all the tested conditions, except the conditions of 30 °C and constant light at 12 °C, the amount of HT-2 toxin increased over three weeks of incubation. Interestingly, the amount of HT-2 toxin decreased under all the conditions between three and four weeks of incubation. The HT-2 toxin production of 36 mg/L was the most efficient at 30 °C after two weeks.

The HT-2-glucoside production was the highest, with 5 mg/L after incubation at 20 °C under constant light for three weeks. Under all the other conditions, except that of 30 °C in the dark, where only 2 mg/L HT-2-glucoside was produced, the amount of HT-2-glucoside produced after four weeks was between 4 mg/L and 5 mg/L.

### 2.3. Determination of the Anomeric State of HT-2-Glucoside

We used flash chromatography for purification in order to determine whether the produced HT-2-glucoside was present in an alpha- or a beta-configuration. To obtain enough material for NMR, we pooled the extracts of the 4-week samples, evaporated the organic solvent, and lyophilized the remaining sample. Subsequently, the redissolved material was fractionated using flash chromatography. The fractions were again analyzed for T-2-toxin, HT-2-toxin, and HT-2-glucoside using the LC-MS/MS multimethod.

Fraction 4, which contained 8305 µg/L HT-2-glucoside, in addition to 95 µg/L HT-2 toxin and 9 µg/L T-2 toxin, was selected for NMR analysis to obtain information about the configuration of the glucosidic bond. For this purpose, the method of choice involved determining the coupling constant of the anomeric proton of the glucose unit: in the alpha-configuration, this proton gives a characteristic *axial*/*equatorial* coupling of *J* = 3–4 Hz, whereas for the beta-configurated glucose unit, a higher coupling constant of *J* = 7–8 Hz is expected due to the *axial*/*axial* orientation (see also [21,22]).

Therefore, the first step was to identify the anomeric protons in fraction 4 through acquisition of a heteronuclear single quantum coherence (HSQC) spectrum (see Supplementary File S1). This experiment showed the presence of two cross-peaks in the characteristic region for the anomeric functions at δ_H_/δ_C_ ca. 4.90/100 ppm. In the second step, the coupling constants of these protons were extracted from the ^1^H NMR spectrum. Both protons had coupling constants of *J* = 4 Hz, proving that the glucosides are in alpha-configuration. As HT-2 toxin has hydroxy groups at C3 and C4, both positions are possible glycosylation sites, which explains why two peaks were observed.

### 2.4. Resistance of HT-2-α-Glucosides against Hydrolysis by Alpha-Glucosidases

A possible toxicologically relevant difference between the alpha-glucosides and beta-glucosides of mycotoxins is that they are expected to be hydrolyzed by different enzymes, alpha-glucosidases or beta-glucosidase, respectively. In humans, salivary alpha amylase is the first alpha-glucosidase that the HT-2-α-glucosides encounter upon ingestion. We therefore tested whether the purified HT-2-α-glucosides could be hydrolyzed by this enzyme. The attempts to separate the two compounds, for which the NMR provided evidence, were unsuccessful, despite the use of a 1.7 µm particle size UHPLC column and a shallow gradient. Nevertheless, the method was used to determine whether the ratio between the HT-2 toxin and HT-2-glucoside was altered by treatment with two different alpha-glucosidases. We employed commercially available salivary amylase as well as the fresh saliva of two male and two female donors and did not obtain evidence of hydrolysis. We also tested for hydrolysis of the HT-2-alpha-glucosides by maltase of baker’s yeast, an enzyme that could act on the glucosides if HT-2-glucoside-contaminated grain was used for brewing or bioethanol production. Again, no evidence of hydrolysis was observed.

## 3. Discussion

In the routine annual screening of *Fusarium* isolates, we came across a *F. sporotrichioides* isolate, which also produced HT-2-glucoside on autoclaved rice. We tried to optimize the production, but the relative amount was low, leading to results that were comparable to those reported previously by Busman [19], with the difference being that those authors set the T-2 toxin as 100%, while we used the sum of the trichothecenes for which standards were available. The available white light conditions did not have a strong effect on T-2 toxin, HT-2 toxin, or HT-2-glucoside production, although it has been reported that inactivation of the components of the velvet complex that transmits the light signal [23] led to a strong reduction in toxin production in other *Fusarium* species (summarized in [24]). In other fungi, it has been reported that the light quality has an influence; for instance, in *Alternaria alternata* red light had no effect, while blue light stimulated alternariol production significantly, yet in case of tenuazonic acid production, no obvious regulation by light was observed [25]. HT-2-glucoside accumulated over time, while one would expect a transient formation if the initially formed glucoside was hydrolyzed again by an alpha-glucosidase (which is involved in the breakdown of the rice matrix rich in starch). This is in agreement with the stability shown against the two tested enzymes.

Busman et al. [19] have shown that two *F. sporotrichioides* isolates—especially on starch containing medium—can produce HT-2-glucoside (and far less T-2-glucoside). In our study, we confirmed HT-2-glucoside formation by an Austrian isolate of this species, but we did not obtain evidence of T-2-glucoside formation, even though the standard of T-2-3-*O*-beta-glucoside was also used in the analytical method. The identification of the actual HT-2-alpha-glucosides in our study with the beta-glucoside standard was due to a short chromatographic protocol in the multi-mycotoxin LC-MS/MS method, and the selection of nonselective fragmentation (604 to 425 due to the loss of glucose and NH4^+^, and HT-2 fragment 323) was rather unspecific. Schmidt et al. [22] used a *Blastobotrys* yeast that converted T-2 toxin (but not HT-2 toxin) into the alpha-glucoside [5], in combination with a deacetylating *Streptomyces* bacterium to generate the HT-2-3-*O*-alpha-glucoside. Although the fragmentation patterns of the alpha- and beta-glucosides were very similar, the authors were able to devise a method for distinction based on different intensities of the ammonium adducts, and they also achieved chromatographic separation between the HT-2-alpha- and HT-2-beta-glucosides. In our study, using NMR, we obtained evidence of the production of two different alpha-glucosides, presumably HT-2-3-*O*- and HT-2-4-*O*-alpha-glucosides. A further (biologically rather unlikely) possibility that is consistent with the mass would be that 3-acetyl-T-2 is first deacetylated at C15 and the glucose is attached to the C15-OH and, subsequently, either C3 (or C4) is deacetylated.

We could not separate the two compounds, despite the use of UHPLC columns. Potentially, the HT-2-glucoside observed by Busman et al. [19] was also a mixture, and the two peaks observed by Lattanzio et al. [26] with retention times of 18.8 min and 19.5 min were alpha- and beta-glucosides of HT-2.

HT-2 toxin has been shown to be metabolized by *Triticum durum* into 23 metabolites [14], for most of which no authentic reference substances are available. Among these metabolites, different glycosylated HT-2 derivates were detected, like HT-2-glucoside or 3-acetyl-HT-2-glucoside [14]. Even though glycosylated metabolites appear to be less toxic, T-2- and HT-2-glucosides have been reported to be hydrolyzed in the pig cecum model, regardless of whether they were present in the alpha- or beta-configuration [27]. Furthermore, HT-2-3-*O*-beta-glucoside can be efficiently hydrolyzed by bacterial beta-glucosidases [28] and also by human gut microbiota in fecal batch culture tests [29].

In the literature, there is a controversy regarding whether the alpha-or beta-glucosides of T-2 and HT-2 toxins are present in naturally contaminated grain and in what ratio. While McCormick et al. [21] and Schmidt et al. [22] provided evidence that the naturally contaminated grain contained alpha-glucosides; Meng-Reiterer et al. and Nathanail et al. [11,30,31] found the beta-glucosides of HT-2 and T-2 in toxin-treated wheat, barley, and oat. These glucosides are difficult to separate, and reference substances are not commercially available. In a recent study, with oat (milling samples) from Scotland [32], T-2-glucoside was the most frequently detected modified mycotoxin (with about 62% positive samples), with ratios ranging from 6 to 154% compared to T-2 toxin. In contrast, using a commercial Austrian oat cultivar (‘Eneko’) treated with T-2 toxin, little T-2-glucoside was found, as it was rapidly deacetylated and then HT-2-glucoside was formed and further metabolized [11], pointing to possible cultivar differences. In the naturally contaminated oat from Scotland, the ratios of HT-2-glucoside to HT-2 toxin ranged from 34 to 174% [32].

Besides their occurrence, an open question remains as to whether the anomeric state is toxicologically relevant. Even under optimized conditions, the HT-2-alpha-glucoside produced in vitro by our fungus is only a marginal fraction of the parental toxin. Unless alpha-glucoside production is much higher *in planta*, the toxicological relevance of the additional ~0.5% HT-2-alpha-glucosides seems negligible. In agreement with previous reports on T-2-alpha-glucoside [21], we obtained negative evidence of hydrolysis of the HT-2-alpha-glucosides by human salivary amylase. Salivary amylase evolved in primates from the pancreatic form (and also independently in other animals), and large copy number differences exist in modern humans [33]. If this enzyme hydrolyzed mycotoxin alpha-glucosides, it could lead to early back-conversion to the parental toxin and (if present in relevant amounts) to different toxicokinetics. We found that the isolated HT-2-alpha-glucosides were resistant to commercial enzyme preparations and also to fresh saliva from donors. Most likely, as described for T-2 alpha-glucoside, our HT-2-alpha-glucosides would also be hydrolyzed in the upper intestinal tract [29]. In contrast to the resistance of the HT-2-alpha-glucoside towards alpha-glucosidases, the HT-2-3-*O*-beta-glucoside is readily hydrolyzed by beta-glucosidase [28], which could be used as a means of distinguishing the anomers present in grain, which have a very similar retention time and fragmentation pattern.

The biosynthetic origin of HT-2-alpha-glucosides or mycotoxin alpha-glucosides in general is another interesting question. Schmidt et al. [22] proposed that the alpha-glucosides they found in contaminated grain were formed “by different glycosyltransferases favoring different glucoside configuration”. Although this cannot be excluded before the identification of the relevant enzymes, this assumption is unlikely as it is in conflict with the enzymatic mechanism of glucosyltransferases. The members of family 1 of UGTs, which are forming small molecule conjugates, are inverting enzymes (http://www.cazy.org/GT1.html, accessed on 1 January 2024), which exclusively form beta-glucosides. Retaining glucosyltransferases (which are mostly involved in complex carbohydrate biosynthesis) exist, but these are typically not conjugating small molecules or, in rare exceptional cases, they have very narrow substrate specificity. For instance, in the retaining family 4 of glucosyltranferases (http://www.cazy.org/GT4.html, accessed on 1 January 2024), an enzyme from *Helicobacter* is able to form cholesteryl-alpha-glucoside [34]. Another rare example is a tetrahydrobiopterin-α-glucosyltransferase from the cyanobacterium *Synechococcus* [35]. As there is broad interest in the biotechnology of such enzymes, a broad specificity alpha-glucosidase would probably not have escaped attention. Instead, a widespread approach to enzymatically synthesizing alpha-glucosides is to employ the transglycosylation activity of (retaining) alpha-glucosidases. For instance, mono-glucosyl derivatives of ginsenoside can be synthesized by transglycosylation with α-glucosidase from rice seeds using maltose as a glucosyl donor [36]. The α-glucoside of tyrosol is produced from starch by α-amylase of *Bacillus subtilis* [37]. The regioselective alpha-glycosylation of hydroxyflavones and hydroxyflavanones is catalyzed by amylosucrase from *Deinococcus geothermalis* [38]. A paper with the misleading title “Characterization of regioselective *glycosyltransferase* of *Rhizobium pusense* JCM 16209^T^ useful for resveratrol 4′-*O*-α-d-glucoside production” actually reports the cloning and utilization of an alpha-glucosidase of this organism [39]. As the equilibrium of the enzymatic reaction is usually on the hydrolysis side, the transglycosylation properties can be improved by site-directed mutagenesis [40].

We propose the following model that can reconcile conflicting findings regarding alpha- and beta-glucosides in cereals. During the early stage of infection, low amounts of toxin diffusing ahead of the invading fungus induce the UGTs of the plant, and beta-glucosides should be present as the predominant metabolite in resistant cultivars. Only beta-glucosides are formed when plants are treated with toxin alone. Yet, when the fungus advances, it can hydrolyze the accumulated beta-glucosides, e.g., with cellulase and cellobiase (as shown for DON-3-*O*-beta-glucoside [41]). During the degradation of starch in heavily diseased or dead kernels, the fungal alpha-glucosidases with high transglycosylation activity, which are still to be identified, could form mycotoxin alpha-glucosides, which might become dominant in heavily contaminated grain. Potentially, starch-degrading enzymes from plants, especially under conditions such as germination and malting, might also contribute to alpha-glucoside formation. Further work is necessary to test this hypothesis and to determinate the ratios in which alpha- and beta-glucosides occur in naturally contaminated grain; this is a question with potential relevance for food and feed safety if higher levels of glycosylated HT-2 are frequently found.

## 4. Conclusions

We isolated a *Fusarium sporotrichioides* strain which produces high levels of T-2 toxin. In addition, HT-2 toxin and HT-2-glucoside were also identified. The upscaling of T-2 toxin production was successful, and the optimal production conditions were also determined. From the extracts, HT-2-glucoside-enriched fractions were obtained via flash chromatography, and two alpha-glucosides could be identified via NMR, leading to the conclusion that HT-2 toxin is glycosylated in both hydroxy groups at C3 and C4. The biosynthetic origin by transglycosylation is proposed, and a model reconciling the different findings regarding the occurrence of the alpha- or beta-glucosides of the HT-2 and T-2 toxins is presented.

## 5. Materials and Methods

### 5.1. Strain Isolation and Secondary Metabolite Profile

In this study, *Fusarium sporotrichioides* 15-39, which was isolated from maize in the district of Tulln in 2015, was used. The fungus was identified using the translation elongation factor EF1-α, which shared 99.56% identity with *F. sporotrichioides* (Accession: MZ078870.1), with a query cover of 98%.

To determine the secondary metabolite profile, 2 g rice was soaked with 2 mL water in a 50 mL tube for one hour and closed with foam plugs before autoclaving. The parboiled long corn rice (Brand Happy Harvest, packaged in Pavia, Italy) which did not contain detectable levels of type A trichothecenes or HT-2-glucoside, was obtained from a local supermarket. The sterile rice was inoculated with a 1 mm^2^ piece of agar and incubated at 20 °C in the dark for two weeks. Subsequently, 8 mL of the extraction solvent (acetonitrile (ACN):H_2_O:acetic acid (HAc), 79:20:1) was added, followed by homogenization using an ULTRA-Turrax (IKA T25, VWR International, Vienna, Austria). The homogenized samples were incubated at 20 °C with shaking (180 rpm) for 1 h, followed by centrifugation. One milliliter of the supernatant was transferred to a 1.5 mL tube. The samples were centrifuged again, and a 1:50 dilution of the supernatant (H_2_O:ACN:HAc, 79:20:1) was prepared for toxin analysis. Analysis was performed using the LC-MS/MS-based multimethod described in Sulyok et al., 2020 [20]. In brief, a 1290 Series HPLC System (Agilent, Waldbronn, Germany) was coupled to a QTrap 5500 LC-MS/MS System (Applied Biosystems SCIEX, Foster City, CA, USA) equipped with a Turbo Ion Spray electrospray ionization source. Chromatographic separation was performed at 25 °C on a Gemini^®^ C18-column, 150 × 4.6 mm i.d., 5 μm particle size, equipped with a C18 4 × 3 mm i.d. security guard cartridge (Phenomenex, Torrance, CA, USA). Confirmation of positive metabolite identification was carried out by the acquisition of two MS/MS signals per analyte in the time-scheduled multiple reaction monitoring mode, which yielded 4.0 identification points, in accordance with the European Commission decision 2002/657. In addition, the retention time and ion ratio had to agree with the related values of the authentic standards within 0.03 min and 30% rel., respectively. Quantitation was based on external calibration using the serial dilutions of a multi-analyte stock solution. The accuracy of the method was verified on a continuous basis by participation in a proficiency testing scheme organized by BIPEA (Gennevilliers, France), with a current rate of z-scores between −2 and 2 of >95% (>1900 results submitted). To determine the percentage of trichothecenes, the sum of all the detected trichothecenes was set as 100%.

### 5.2. Optimization of T-2 Toxin Production Conditions

To find the optimal conditions for T-2 toxin production, 10^5^ spores of *F. sporotrichioides* were inoculated on a 10 g autoclaved rice medium (baby food jars, vented lids) and incubated at 12 °C, 16 °C, 20 °C, and 30 °C in the dark, as well as at 12 °C and 20 °C under 24 h light (plant growth chambers with Osram Lumilux cool white 840 lamps). No 16 °C room with light was available; therefore, for this temperature only incubation in the dark was performed. The experiment was carried out over a total time period of 4 weeks. Every week, three samples from each temperature and condition were transferred to −20 °C until they were all extracted.

Forty milliliters of extraction buffer (acetonitrile:water:acetic acid, 79:20:1) was added to each culture; the rice was mixed with a spatula and the cultures were homogenized using an ULTRA-Turrax. Extraction was performed by shaking the homogenates at 180 rpm at 20 °C for 1 h. After extraction, the homogenates were centrifuged for 10 min at 20 °C at 4000 rpm. One milliliter of supernatants was transferred to 1.5 mL tubes and centrifuged for 10 min at 14,000 rpm (RT), and 100 µL of the supernatants was transferred to 900 µL of dilution buffer (water + acetonitrile + acetic acid (79:20:1)) in HPLC vials. Six hundred microliters of the undiluted extracts were also transferred to HPLC vials and stored at 4 °C. The remaining extracts were stored at −20 °C until purification.

### 5.3. Isolation of HT-2-Glucoside from Crude Extract (Flash Chromatography)

The solvent of the pooled crude extracts was evaporated, followed by lyophilization of the samples. The dried powder (≈250 mg) was mixed with 15 g of Celit^®^ 545 (Carl Roth, Karlsruhe, Germany) and dried under forced air at atmospheric pressure. Then, the whole sample was applied to a reversed-phase silica gel vacuum flash chromatograph (Interchim, puriFlash^®^450, Montluçon, France), using two superimposed Interchim puriFlash^®^ 32 g silica C18-IR-50C18-F0025 flash columns (particle size: 50 µm). The columns were eluted with a two-solvent gradient (solvent A: H_2_O, solvent B: ACN). The starting linear gradient from 10% B to 100% B in 45 min at a flow rate of 15 mL/min was followed by an isocratic gradient of B at 100% B for a further 20 min. The UV 254 nm and UV scan 200–400 nm modes were used for the detection and final separation of 11 main peak fractions (F1-F11, Table 1), which were consequently sampled (100 µL aliquots) and diluted at a ratio of 1:50 for LC-MS/MS analysis. The target compound, HT-2-glucoside, was found in fraction F4 by eluting at 20–24 min (61 mL with yield ≈ 500 µg of HT-2-glucoside, with traces of HT-2 toxin and T-2 toxin, 5.8 µg and 0.6 µg, respectively (Table 1)). The dried fraction F4 was then investigated by nuclear magnetic resonance spectroscopy (NMR) for the presence of alpha- or beta-configurated HT-2-glucoside.

### 5.4. NMR Analysis of HT-2-Glucosides

The NMR spectra were recorded on a Bruker Avance II 400 (resonance frequencies 400.13 MHz for ^1^H and 100.61 MHz for ^13^C) equipped with a 5 mm N_2_-cooled broadband observe cryoprobe head (Prodigy), with z–gradients at room temperature with standard Bruker pulse programs. The sample was dissolved in 0.6 mL of D_2_O (99.8% D, Eurisotop, Saint-Aubin, France). Edited HSQC experiment was acquired with 1 k × 256 data points using adiabatic pulse for the inversion of the ^13^C and GARP sequence for broadband ^13^C decoupling, optimized for ^1^*J*_(CH)_ = 145 Hz.

### 5.5. Alpha-Glucosidase Hydrolysis Experiments

To test the ability of human salivary amylase to hydrolyze HT-2-alpha-glucosides, 1000 units of this lyophilized enzyme were obtained from Sigma (A1031-1KU), and a solution was prepared (2 U/µL). In a 50 µL reaction volume, 5 µL 10× buffer (500 mM Tris.Cl pH7, 500 mM NaCl, 10 mM CaCl_2_), 5 µL enzyme (2 U/µL and serial dilutions thereof), and 5 µL toxin dilution (giving 7 mg/L HT-2-alpha-glucoside and 2% methanol in the final reaction volume) were combined and incubated at 37 °C for 2 h. Likewise, fresh saliva from 2 male and 2 female volunteers was obtained (with informed consent). The saliva was diluted with water at a ratio of 1:2 to reduce viscosity, and 40 µL was combined with 5 µL 10× buffer and 5 µL toxin solution. The reactions were stopped by the addition of 50 µL methanol, and the enzyme precipitate was removed by centrifugation for 10 min (14,800 rpm in an Eppendorf centrifuge). The transferred supernatant was incubated at −20° and centrifuged again; the supernatant was transferred to HPLC vials with micro-inserts. Similarly, maltase from yeast (alpha-glucosidase) was obtained from Sigma (G5003-100UN) and dissolved in 200 µL. As with the saliva, 40 µL of this enzyme solution (1:2 dilutions thereof) was combined with 5 µL 10× buffer and 5 µL toxin and incubated for 2 h.

The hydrolysis experiments were measured on an Agilent 1290 UHPLC system coupled to a Sciex 6500+ QqQ mass spectrometer. A Phenomenex Kinetex Evo C18 column (2.1 × 150 mm; 1.7 µm particle size) at 25.0 °C was used for separation. Ultrapure water (mobile phase A) and ACN (mobile phase B) were both modified with 0.1% formic acid and 10 mM ammonia solution and delivered at a flow rate of 300 µL/min. Ten percent B was held for 3 min, before a linear gradient to 40% B was applied till 16 min. After a 4 min wash with 100% B, the column was re-equilibrated for 5 min till the end of the run at 25 min. A valve directed the eluent into the mass spectrometer between 5 and 15 min, and 2.00 µL of the samples was injected. A Turbo Spray IonDrive electrospray source was operated at 550 °C and 5500 V for ionization in a positive ion mode. The ion source gas settings were 50 psi each, while 30 psi of curtain gas was used. Selected reaction monitoring (SRM) was applied with a 20 ms dwell time to detect the ammonium adducts of the HT-2-glucosides and HT-2 toxin as well as the possible hydrolysis product T-2 triol. The following SRM settings were used: HT-2-glucosides: *m*/*z* 604.3 > 157.0 (declustering potential (DP) 86 V, collision energy (CE) 36 eV), 604.3 > 105.0 (CE 68 eV), 604.3 > 323.2 (CE 17 eV), 604.3 > 425.2 (CE 17 eV); HT-2 toxin: *m*/*z* 442.3 > 263.1 (DP 76 V, CE 21 eV), 442.3 > 215.1 (CE 24 eV); T-2 triol: *m*/*z* 400.3 > 281.1 (DP 71 V, CE 13 eV), 400.3 > 215.1 (CE 17 eV). The LC-MS/MS system was operated using Sciex OS 3.0. T-2 triol eluted at 6.60 min; HT-2 toxin eluted at 12.11 min; and the HT-2-glucosides eluted at 12.90 min.

## Figures and Tables

**Table 1:** Fractions (F1–F11) from flash chromatography with concentrations of T-2 toxin, HT-2 toxin, and HT-2-glucoside, as detected and quantified by LC-MS/MS; <LOD, limit of detection; * fraction used for NMR analysis (with highest amount of HT-2-glucoside).

Fraction Nr.	Elution Time (min.)	Volume (mL)	Metabolites Detected (µg/L)		
			HT-2- Glucoside	HT-2 Toxin	T-2 Toxin
1	3–8	75	<LOD	<LOD	<LOD
2	9–12	85	<LOD	<LOD	<LOD
3	14–15	86	<LOD	<LOD	1
4 *	20–24	61	8305	95	9
5	24–27	55	136	127,650	44
6	28–30	56	<LOD	1482	191,500
7	31–33	39	<LOD	1114	137,600
8	34	32	<LOD	287	122,700
9	36–39	59	<LOD	155	12,800
10	40	92	<LOD	79	4554
11	45–51	156	<LOD	75	3307

## Data Availability

Data is contained within the article or supplementary material.

## Supplementary Materials

The following supporting information can be downloaded at: https://www.mdpi.com/article/10.3390/toxins16020099/s1, File S1 (NMR data) and Figure S1 (Scheme—Biosynthesis of type A trichothecenes).
