# Supplementary material for: Fusarium sporotrichioides Produces Two HT-2-α-Glucosides on Rice
Source: Toxins (Basel). 2024 Feb 10;16(2):99. doi: 10.3390/toxins16020099 (PMC10893509; doi:10.3390/toxins16020099)
Supplement: Supplementary file 1 [file toxins-16-00099-s001.zip › toxins-2837861-supplementary/Supplementary File 1.pdf]

bimm-502-ht2-toxinglucoside
T = 323 K

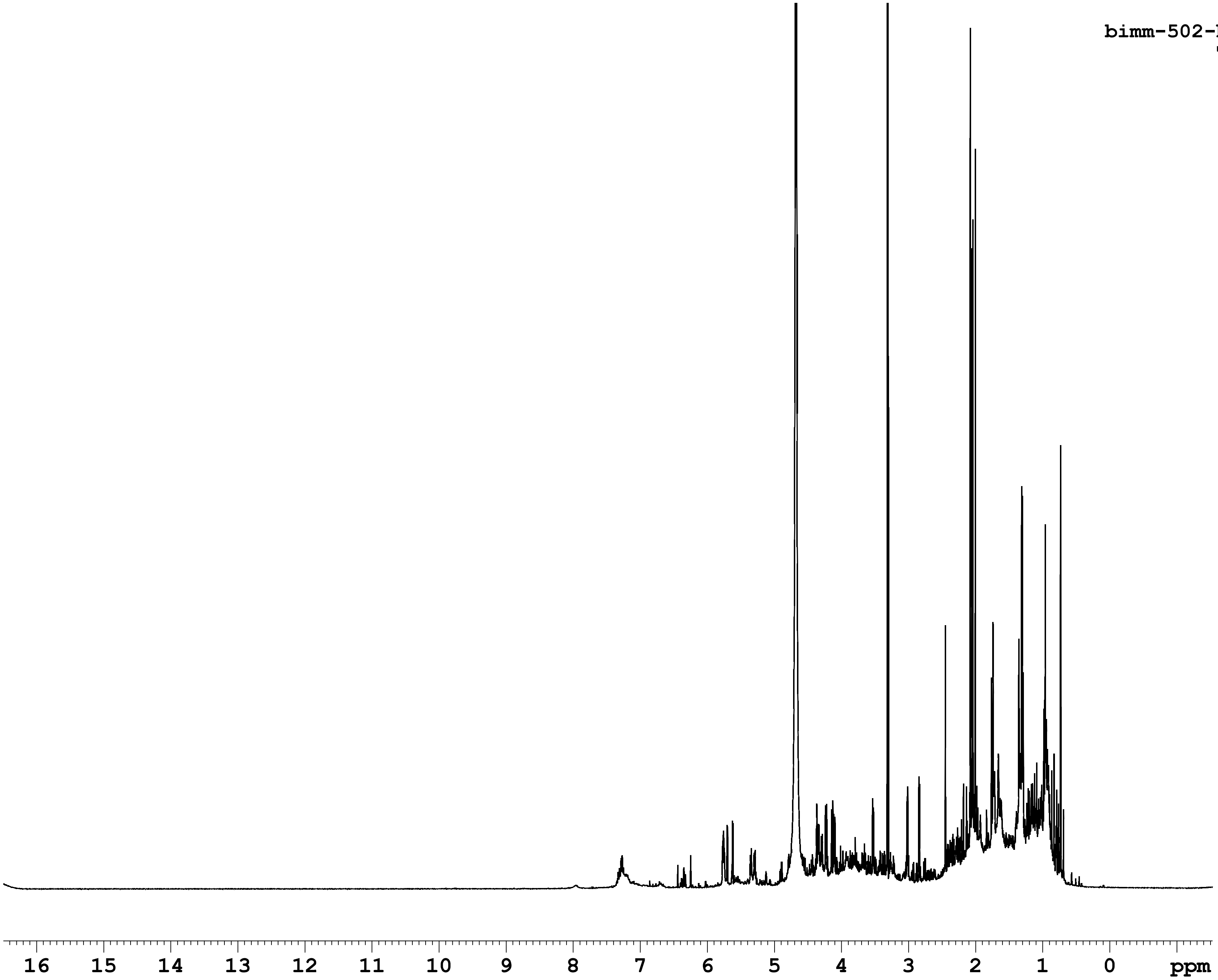

Current Data Parameters
NAME bimm-502-ht2-toxinglucoside
EXPNO 3
PROCNO 1

F2 - Acquisition Parameters
Date_ 20231025
Time 16.06
INSTRUM spect
PROBHD 5 mm CPPBBO BB
PULPROG zg30
TD 32768
SOLVENT MeOD
NS 16
DS 0
SWH 7211.539 Hz
FIDRES 0.220079 Hz
AQ 2.2719147 sec
RG 45.2
DW 69.333 usec
DE 10.00 usec
TE 323.0 K
D1 1.00000000 sec
TD0 1

======== CHANNEL f1 ========
SFO1 400.1330010 MHz
NUC1 1H
P1 11.77 usec
PLW1 9.00000000 W

F2 - Processing parameters
SI 32768
SF 400.1300075 MHz
WDW GM
SSB 0
LB -0.30 Hz
GB 0.3
PC 1.00

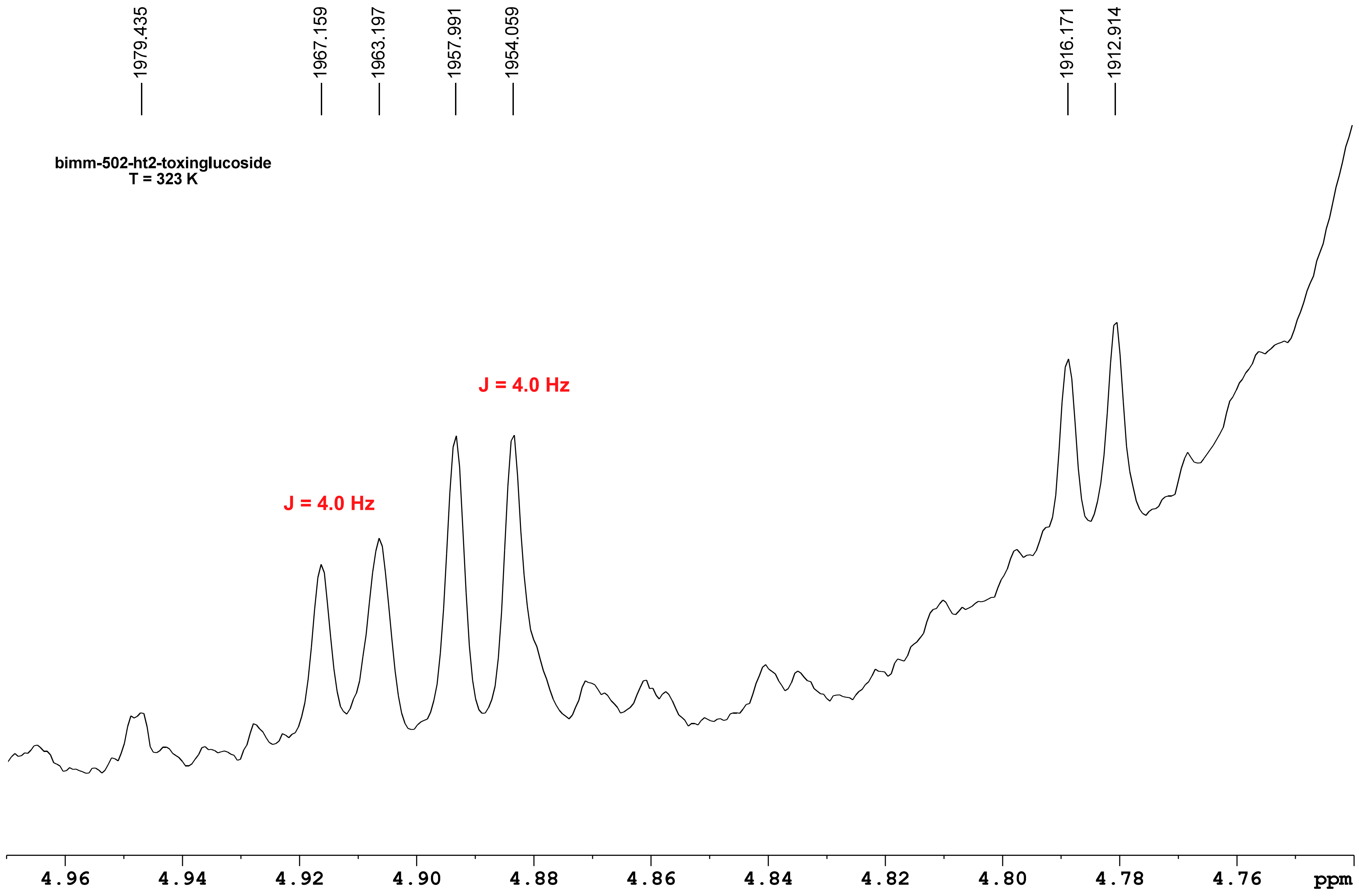

1979.435
1967.159
1963.197
1957.991
1954.059
1916.171
1912.914
bimm-502-ht2-toxinglucoside
T = 323 K
J = 4.0 Hz
J = 4.0 Hz
4.96
4.94
4.92
4.90
4.88
4.86
4.84
4.82
4.80
4.78
4.76
ppm

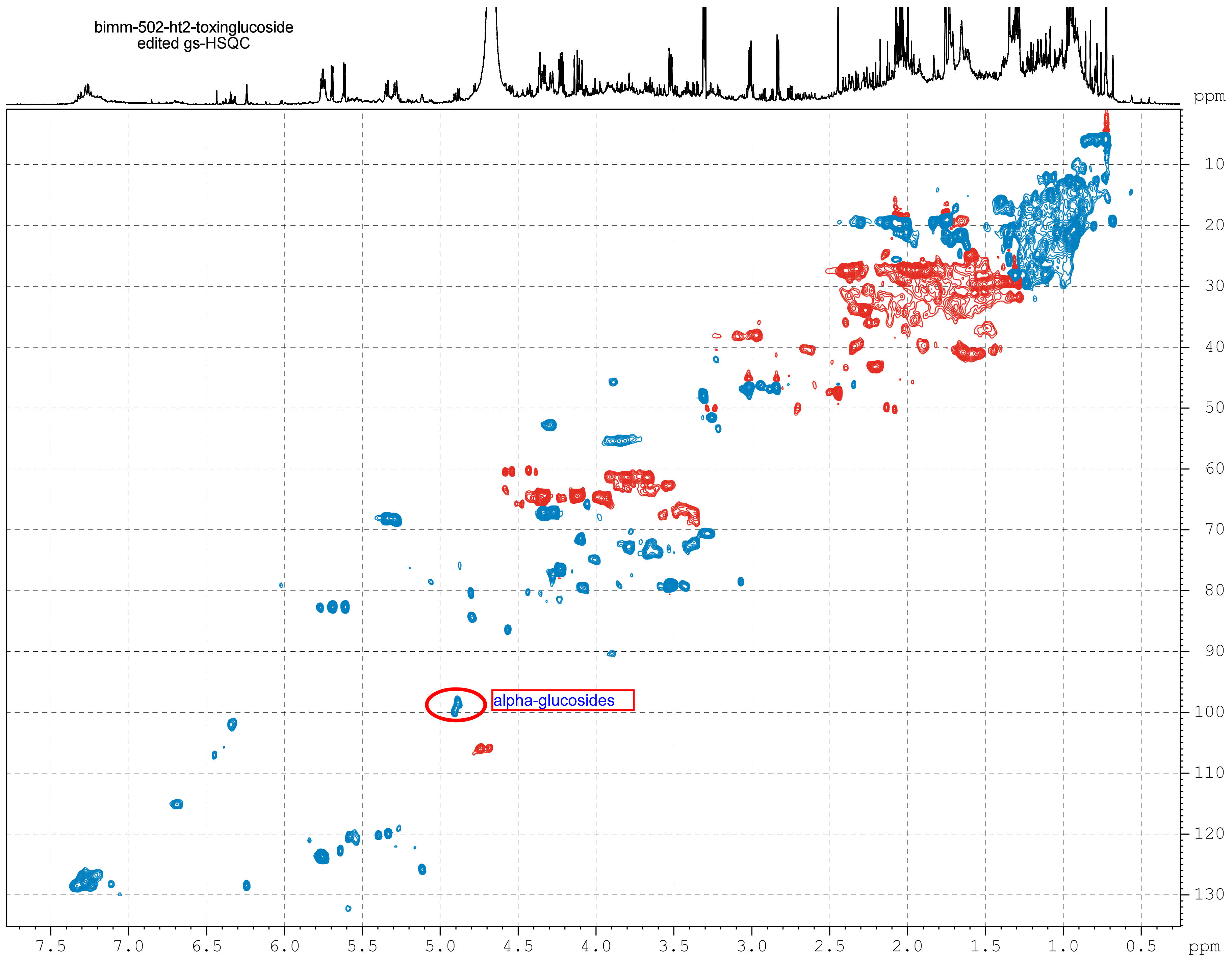

Current Data Parameters
NAME bimm-502-ht2-toxinglucoside
EXPNO 2
PROCNO 1

F2 - Acquisition Parameters
Date_ 20231025
Time 15.35
INSTRUM spect
PROBHD 5 mm CPPBBO BB
PULPROG hsqcedetgpsp.3
TD 1024
SOLVENT MeOD
NS 8
DS 16
SWH 4795.396 Hz
FIDRES 4.683004 Hz
AQ 0.1067691 sec
RG 2050
DW 104.267 usec
DE 10.00 usec
TE 303.0 K
CNST2 145.0000000
D0 0.00000300 sec
D1 1.00000000 sec
D4 0.00172414 sec
D11 0.03000000 sec
D16 0.00020000 sec
D21 0.00360000 sec
IN0 0.00003310 sec

======== CHANNEL f1 ========
SFO1 400.1324008 MHz
NUC1 1H
P1 11.77 usec
P2 23.54 usec
P28 1000.00 usec
PLW1 9.00000000 W

======== CHANNEL f2 ========
SFO2 100.6203150 MHz
NUC2 13C
CPDPRG[2 garp
P3 10.20 usec
P14 500.00 usec
P31 2119.00 usec
PCPD2 65.00 usec
PLW0 0 W
PLW2 32.00000000 W
PLW12 0.78799999 W
SPNAM[3] Crp60,0.5,20.1
SPOAL3 0.500
SPOFFS3 0 Hz
SPW3 5.08680010 W
SPNAM[18 Crp60_xfilt.2
SPOAL18 0.500
SPOFFS18 0 Hz
SPW18 0.97992998 W

====== GRADIENT CHANNEL =====
GPNAM[1] SMSQ10.100
GPNAM[2] SMSQ10.100
GPZ1 80.00 %
GPZ2 20.10 %
P16 1000.00 usec

F1 - Acquisition parameters
TD 128
SFO1 100.6203 MHz
FIDRES 236.027191 Hz
SW 150.126 ppm
FnMODE Echo-Antiecho

F2 - Processing parameters
SI 2048
SF 400.1300094 MHz
WDW QSINE
SSB 2
LB 0 Hz
GB 0
PC 1.40

F1 - Processing parameters
SI 1024
MC2 echo-antiecho
SF 100.6127690 MHz
WDW QSINE
SSB 2
LB 0 Hz
GB 0

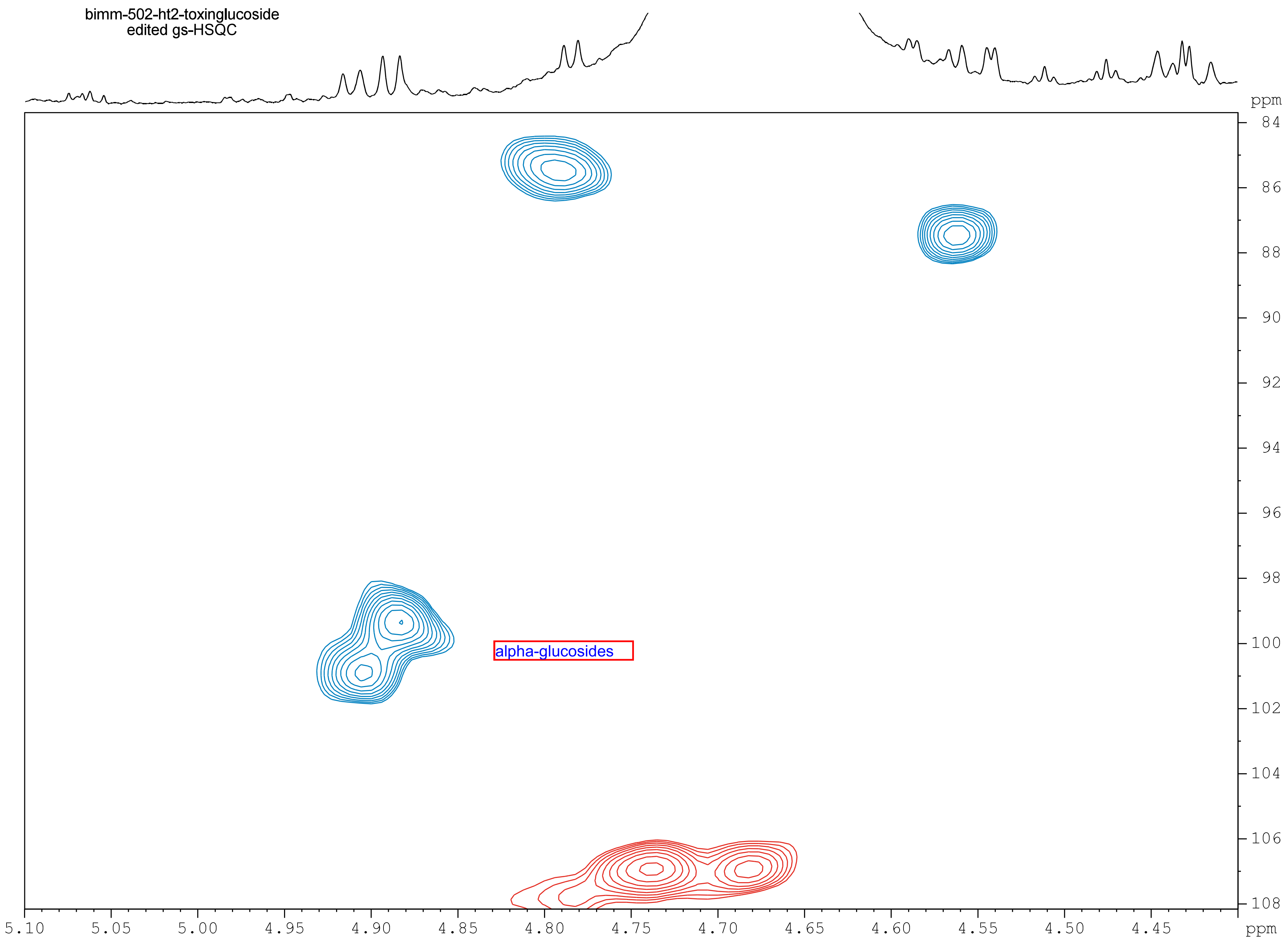
bimm-502-ht2-toxinglucoside
edited gs-HSQC
alpha-glucosides
ppm
84
86
88
90
92
94
96
98
100
102
104
106
108
5.10
5.05
5.00
4.95
4.90
4.85
4.80
4.75
4.70
4.65
4.60
4.55
4.50
4.45
ppm
