# Supplementary figures and images for: Fusarium sporotrichioides Produces Two HT-2-α-Glucosides on Rice
Source: Toxins (Basel). 2024 Feb 10;16(2):99. doi: 10.3390/toxins16020099 (PMC10893509; doi:10.3390/toxins16020099)

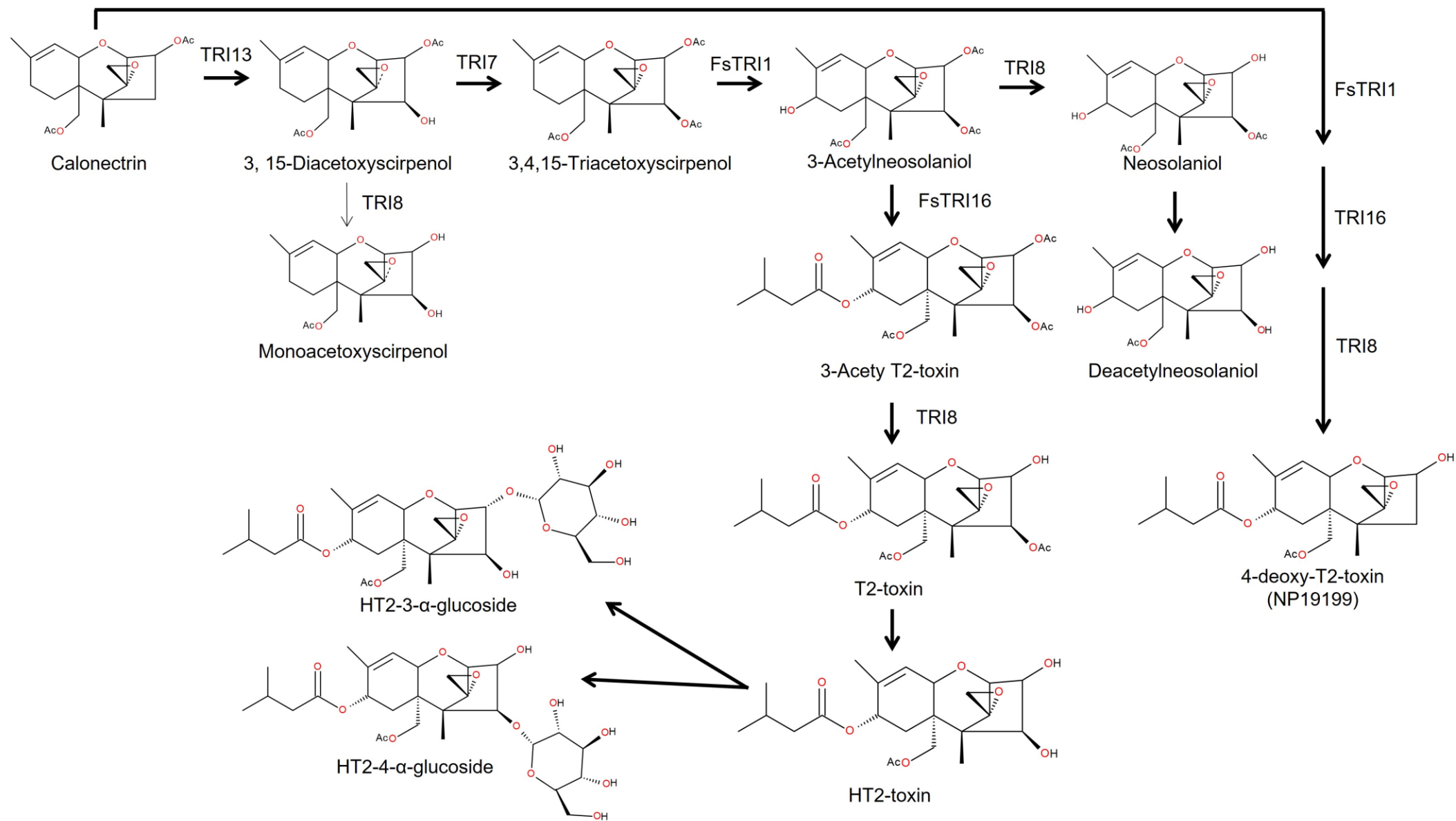

Fig. S1

Supplement: Supplementary file 1 [file toxins-16-00099-s001.zip › toxins-2837861-supplementary/Fig S1.pdf]
